# VISTA expression and patient selection for immune-based anticancer therapy

**DOI:** 10.3389/fimmu.2023.1086102

**Published:** 2023-02-20

**Authors:** Alexander S. Martin, Michael Molloy, Andrey Ugolkov, Reinhard W. von Roemeling, Randolph J. Noelle, Lionel D. Lewis, Melissa Johnson, Laszlo Radvanyi, Robert E. Martell

**Affiliations:** ^1^ Division of Hematology/Oncology, Tufts Medical Center, Boston, MA, United States; ^2^ ImmuNext Inc., Lebanon, NH, United States; ^3^ Curis Inc., Lexington, MA, United States; ^4^ Department of Microbiology and Immunology, Norris Cotton Cancer Center Geisel School of Medicine at Dartmouth, Lebanon, NH, United States; ^5^ Sarah Cannon at Tennessee Oncology, Nashville, TN, United States; ^6^ Ontario Institute for Cancer Research, Toronto, ON, Canada

**Keywords:** VISTA, immune checkpoint, cancer, biomarkers, cancer immunotherapy, tumor immunity, tumor microenvironment

## Abstract

V-domain Ig suppressor of T-cell activation (VISTA) is a B7 family member that plays key roles in maintaining T cell quiescence and regulation of myeloid cell populations, which together establish it as a novel immunotherapy target for solid tumors. Here we review the growing literature on VISTA expression in relation to various malignancies to better understand the role of VISTA and its interactions with both tumor cells and immune cells expressing other checkpoint molecules within the tumor microenvironment (TME). The biology of VISTA creates several mechanisms to maintain the TME, including supporting the function of myeloid-derived suppressor cells, regulating natural killer cell activation, supporting the survival of regulatory T cells, limiting antigen presentation on antigen-presenting cells and maintaining T cells in a quiescent state. Understanding these mechanisms is an important foundation of rational patient selection for anti-VISTA therapy. We provide a general framework to describe distinct patterns of VISTA expression in correlation with other known predictive immunotherapy biomarkers (programmed cell death ligand 1 and tumor-infiltrating lymphocytes) across solid tumors to facilitate investigation of the most efficacious TMEs for VISTA-targeted treatment as a single agent and/or in combination with anti-programmed death 1/anti-cytotoxic T lymphocyte antigen-4 therapies.

## Introduction

The expression of immune checkpoint molecules on the surface of cells is crucial for self-tolerance, which prevents the immune system from attacking various normal and foreign cells indiscriminately (1). Malignant tumors have been known to harness these immune checkpoint molecules to evade detection and clearance by circulating immune effector cells.

With the discovery of immune checkpoint inhibitors (CPIs), many patients with advanced-stage solid cancers, including melanoma, lung cancer, hepatocellular carcinoma, and bladder cancer have experienced dramatic improvements in antitumor efficacy over the last two decades (2–5). Cancer patients eligible for immunotherapies that target these checkpoint molecules have increased from an estimated 2% in 2011 to nearly 44% in 2018, with the estimated response to these therapies increasing from approximately 0.1% to 13%, respectively (6).

VISTA (V-domain Ig suppressor of T-cell activation) is a novel checkpoint molecule in the B7 family that uniquely impacts cancer immune evasion due to its expression patterns and functions. Unlike checkpoints that primarily regulate T-cell effector function and exhaustion, VISTA plays multiple roles in supporting the function of myeloid-derived suppressor cells (MDSCs), regulating natural killer (NK)-cell activation, supporting the survival of regulatory T cells, limiting antigen presentation on antigen-presenting cells (APCs), and also maintaining T cells in a quiescent state (7, 8). Preclinical work has identified VISTA as a promising target for anticancer therapeutic development, and several candidates are near or have already entered early clinical development. This review explores VISTA expression and function to understand potential predictive markers for anti-VISTA tumor efficacy.

Determining how to select solid tumors with the best likelihood to respond to a single or combination of CPIs has been challenging. Recently, there has been much interest in looking broadly at the tumor microenvironment (TME – comprising tumor cells, immune cells, and stromal cells) for predictive expression patterns. Several predictive markers have emerged from this work, including programmed death-ligand 1 (PD-L1) expression, cytotoxic T-cell infiltration, tumor mutational burden (TMB) and microsatellite instability (9–11). Based on understanding checkpoint molecule expression in the TME as a potential predictive marker of response to certain immunotherapies, this article focuses on the novel immune checkpoint molecule, VISTA. It further discusses the patterns of its expression in the different cell types along with other parameters within the TME (12). We describe the current understanding of VISTA biology in the context of these potential predictive patterns and provide insights into how VISTA may play a role in regulating antitumor responses in different types of immune tumor microenvironments alone or together with other checkpoint molecules.

## Structure of VISTA

VISTA, also known as c10orf54, VSIR, SISP1, B7-H5, PD-1H, DD1α, Gi24, and Dies1, is a Type I transmembrane protein comprising a single N-terminal immunoglobulin (Ig) V-domain, a stalk of approximately 30 amino acids (aa), a transmembrane domain, and a 95-aa cytoplasmic tail (5). The 3D structure of the human VISTA extracellular domain (ECD) has been published by multiple groups (5, 13–16); it consists of an immunoglobulin IgV domain and a stalk region that links to the transmembrane domain. The *VISTA* gene is located within the intron of the *CDH23* gene on human chromosome 10 (location q22.1), far from the cluster that contains other B7 superfamily members (17–19). In the CNS, *VISTA* expression appears to be independent of *CDH23* as shown by a reduction in *VISTA* expression in acutely isolated microglia 3 hrs after LPS injection while *CDH23* expression remained unchanged (20).

VISTA is the most conserved among the B7 members and shows 90% homology between mouse and human. Unlike conventional B7 family members, the intracytoplasmic domain of VISTA does not contain ITAM, ITIM, or ITSM sequences, indicating that regulation of VISTA-involved signaling pathways differs from other B7 family molecules programmed cell death-1 (PD-1), PD-L1, cluster of differentiation (CD)28 and cytotoxic T lymphocyte antigen 4 (CTLA-4). Instead, VISTA’s cytoplasmic domain contains several motifs, including Src homology domain2 (SH2) binding motif (YxxQ), as well as multiple casein kinase 2 and phosphokinase C phosphorylation sites. The exact role of these intracellular signaling moieties in driving immune suppressive activities is unknown.

**Figure 1:**
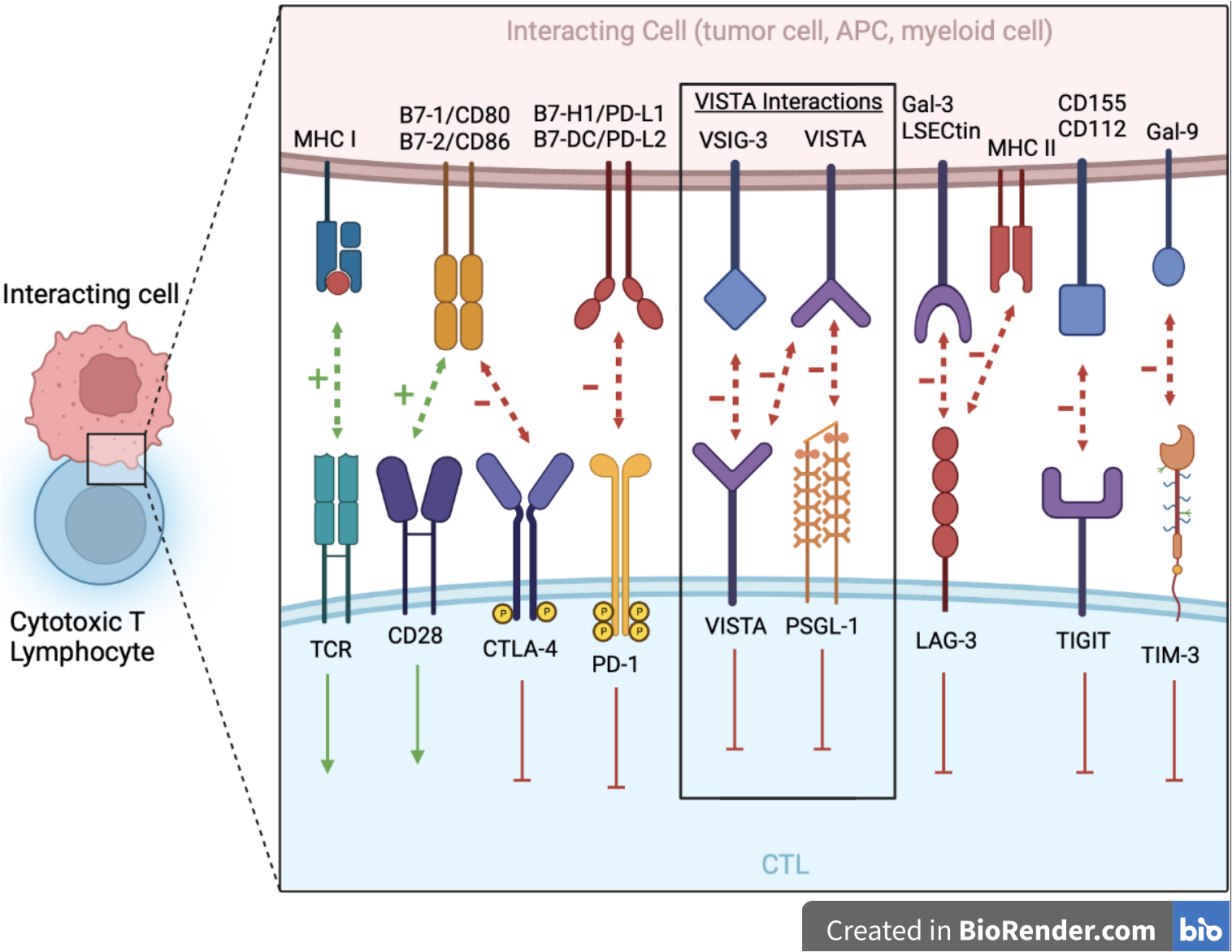
The major NCRs in the Ig superfamily are shown at the junction between the CTL and interacting cell (e.g., tumor cell, APC, myeloid cell, etc.). VISTA serves dual immunosuppressive roles as both a ligand on myeloid cells/APCs with PSGL-1 being its receptor on CTLs and a receptor on CTLs with VSIG-3 as its ligand. Additionally, there is potential for VISTA–VISTA homotypic interaction. Blocking antibodies toward these targets is showing great promise in immunotherapy. APCs, antigen presenting cell; CTL, cytotoxic T lymphocyte; Gal-3, galectin-3; Gal-9, galectin-9; LAG-3, lymphocyte activation gene 3; LSECtin, liver and lymph node sinusoidal endothelial cell C-type lectin; MHC I, major histocompatibility complex class I; MHC II, major histocompatibility complex class II; NCR, negative checkpoint receptors; PSGL-1, P-selectin glycoprotein ligand 1; TCR, T cell receptor; TIGIT, T cell immunoglobulin and immunoreceptor tyrosine-based inhibitory motif domains; TIM-3: T cell immunoglobulin mucin-3; TME, tumor microenvironment; VSIG-3, V-set and Ig domain-containing 3.

The ligands binding VISTA are under active investigation, and no unique ligand has been clarified. It could be that different ligands may engage VISTA under different conditions. Two VISTA binding partners under investigation: one being a known receptor on T cells, P-selectin glycoprotein ligand 1 (PSGL-1), and a second being V-set and Ig domain-containing 3 (VSIG-3), a known cell surface adhesion molecule with increased expression in GI tumors, might indicate dual roles of VISTA as both a ligand for PSGL-1 on T cells and as a receptor for VSIG-3 on tumor cells or myeloid cells (**Figure 1**) (14, 21). This dual functionality may explain the differences in VISTA expression and timing of expression on different cells in the TME, including immune cells and tumor cells. In addition, a VISTA–VISTA homotypic interaction is possible. Multiple histidine residues along the rim of the VISTA extracellular domain mediate binding to the adhesion and coinhibitory receptor, PSGL-1, on tumor-infiltrating lymphocytes with low expression on B cells (22). VSIG‐3 inhibits human T‐cell proliferation in the presence of T‐cell receptor signaling. VSIG‐3 significantly reduces cytokine and chemokine production by human T cells, including IFN‐*γ*, IL‐2, IL‐17, CCL5/Rantes, CCL3/MIP‐1*α*, and CXCL11/I‐TAC. Anti‐VISTA neutralization antibodies attenuate the binding of VSIG‐3 and VISTA and cause VSIG‐3‐induced T‐cell inhibition (21). One study compared VISTA binding to VSIG-3 versus PSGL-1 at a neutral pH of 7.4, which showed VISTA binding to VSIG-3 at 20 nM but no detectable binding to PSGL-1 (14). This interaction between PSGL-1 and VISTA appears to be pH mediated with environmental pH ≤6.0 favoring binding, which is often the case within the TME.

Recently, V-Set and Immunoglobulin domain-containing 8 (VSIG-8) has been discovered to be another potential binding partner for VISTA (23). A VSIG-8 inhibitor, L557-0155, which inhibits VISTA binding to VSIG-8 promoted cytokine production, including TNF-α and IFN-γ, as well as cell proliferation in peripheral blood mononuclear cells and suppressed melanoma growth. Additionally, the monocyte heparan sulfate proteoglycan Syndecan-2 (Sdc2-HSPG) has been identified as a novel regulator of VISTA binding to monocytic cells. Both Sdc2 expression and modification with HSPG (Sdc2-HSPG) are critical for monocyte migration, chemotaxis, and maturation (24).

The intracellular signaling of VISTA is not yet clearly defined, but VISTA’s binding partners have signaling potential as well, which would indicate it can function as a ligand for PSGL-1 and/or VSIG-3. PSGL-1 mediates leukocyte trafficking by binding to selectins, which involves adaptor proteins DNAX-activating protein of 12 kDa (DAP12) and Fc receptor γ (FcRγ), ezrin, radixin, and moesin (ERM) proteins, and Src and Syk kinases (25). VSIG3’s ECD contains an N-terminal IgV-like domain and IgC-like domain and a cytoplasmic domain with a C-terminal PDZ-binding motif that might interact with cytoplasmic scaffolding proteins containing a PDZ domain. VSIG3 can function as an adhesion molecule that regulates synaptic transmission and plasticity by binding to the postsynaptic scaffolding protein PSD-95 (21). Unlike VSIG-3, which is primarily expressed in non-hematologic tissues (testis and ovary with lower amounts in the brain, kidney, and skeletal muscle), PSGL-1 is primarily expressed in hematopoietic cells (26). Having various binding partners and environmental acidities at which VISTA binds to these receptors may give insights into how VISTA provides negative immune regulation in specific tissues.

Discovering specific patterns of VISTA expression within the TME may offer further insights into its tumor growth-enhancing role and illuminate which tumors anti-VISTA agents will be most responsive to VISTA blockade.

## Expression of VISTA in human cells and tissues

### VISTA expression during steady-state conditions

VISTA protein expression, as measured by immunohistochemistry (IHC), is seen on the cell membrane since it is a transmembrane protein; however, there is often some degree of cytoplasmic expression as well, which may be due to intracellular stores of VISTA which are transported to the cell membrane after an appropriate stimulus (27). Intra-cellularly, VISTA has been found to predominantly localize within endosomal compartments, as evidenced by its colocalization with Ras-related protein (Rab-11) (17). VISTA is highly expressed on human hematopoietic cells and at the highest densities in the myeloid lineage (monocytes, macrophages, dendritic cells) based on flow cytometry. Significantly higher densities of VISTA have been observed in CD14+ monocytes and are upregulated by certain cytokines and TLR ligands (12). VISTA is expressed to a lesser extent in CD66b+ neutrophils, followed by naïve CD4+ and CD8+ T cells and regulatory Foxp3+ T cells (12, 18, 28). Notably, there is minimal to no VISTA expression on resting CD19+ B cells (12). Both lymphoid CD11cloCD123+HLA-DR+ and myeloid CD11c+CD123loHLA-DR+ subsets of dendritic cells expressed VISTA as well (12).

**Figure 2:**
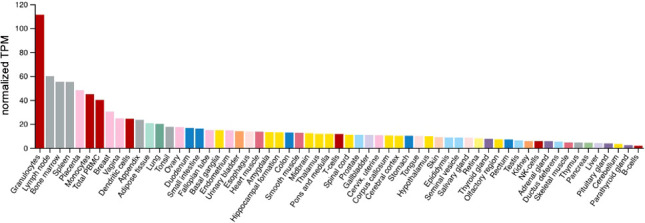
*VISTA* expression in normal tissues/cells. Y axis: Consensus normalized expression in transcripts per million (TPM) for 55 tissue types, created by combining the data from the three transcriptomics datasets. Color coding is based on tissue groupings with common functional features. HPA, Human Protein Atlas; GTEx, Genotype-Tissue Expression project and FANTOM5.

*VISTA* mRNA is also expressed in other normal tissues, as depicted in **Figure 2**. Using quantitative reverse transcription polymerase chain reaction performed on a TissueScan human normal cDNA array using *VISTA* and *PD-L1* Taqman assays, the highest relative *VISTA* expression was seen in the placenta followed by the spleen, leukocytes, lung, lymph node, uterus, bone marrow, fat tissue, and trachea (12). These findings are similar to *VISTA* expression found in The Human Protein Atlas (HPA) (http://www.proteinatlas.org), which combines RNA-seq data on 55 tissue types and six blood cell types taken from three large transcriptomics datasets (HPA, Genotype-Tissue Expression project, and FANTOM5) using the internal normalization pipeline. After blood, bone marrow and lymphoid tissue, the next highest specific tissue expression in descending order was noted in the placenta, breast, vagina, appendix, adipose tissue, lung, tonsil, ovary, duodenum, and small intestine (**Figure 2**) (29).

### VISTA expression in inflammatory states

VISTA expression differs in inflammatory conditions compared to the steady-state conditions described above. In human CD14+ monocytes, VISTA expression was significantly upregulated by the cytokines IL-10 and IFN-γ and agonists of TLR3 (Poly I:C) and TLR5 (flagellin), but was not affected by ligands of TLR1, TLR2, TLR4 and was downregulated by TLR8/9 (28), which suggests VISTA is involved in innate immunity to viral and bacterial pathogens. For chronically-infected HIV patients where chronic immune activation is prevalent, VISTA expression is two- to four-fold higher on activated monocytes compared to seronegative controls and is associated with spontaneous cytokine expression by HIV-specific T cells (28). These results suggest that VISTA might play a functional role in modulating immune activation and immune response in HIV infection. However, *in vivo* mouse models show that VISTA expression is strongly downregulated on antigen-specific CD4+ T cells under certain inflammatory conditions (e.g., stimulation by LPS, CFA, and poly-IC) in which CTLA-4, LAG3, and PD-1 play prominent immunoregulatory roles (7). Interestingly, a VISTA agonist monoclonal antibody prevented acute graft versus host disease (GVHD) in semi- and fully-allogeneic murine models, leading to full chimerism following treatment (18). Additionally, in a murine model of multiple sclerosis, antibody-mediated VISTA blockade exacerbated the development and disease severity of experimental autoimmune encephalomyelitis (5), which indicates an inhibitory role for the VISTA ligand *in vivo*. While VISTA expression in the CNS is normally highest in microglia, VISTA staining was almost absent on the microglia in human chronic multiple sclerosis lesions (20). Together these findings show both the importance and variability of VISTA expression during inflammatory conditions.

## Expression of VISTA in aggregate malignant tumor samples

Analyses of *VISTA* expression in 31 different malignant tumor types using RNA-seq from The Cancer Genome Atlas (TCGA) pan-cancer samples revealed the highest normalized *VISTA* expression was observed in mesothelioma, low-grade glioma (LGG), kidney renal clear cell carcinoma (KIRC), pancreatic adenocarcinoma (PAAD), head and neck squamous cell carcinoma (HNSCC), sarcoma (SARC), and glioblastoma (GBM) (30). The Human Protein Atlas analysis of just 17 tumor types from the pan-cancer samples of the TCGA, excluding mesothelioma and sarcoma, revealed similar results. Specific analysis of the TCGA Pan-Cancer Atlas RNA-seq dataset controlling for leukocyte infiltration in samples indicated that *VISTA* expression on tumor cells specifically was most common in GBM, HNSCC, KIRC, LGG, MESO, SARC (31). An additional analysis of TCGA pan-cancer samples compared mRNA *VISTA* expression of malignant tumors with their paired normal tissue samples from the GTEx database. There was a significant increase in *VISTA* expression in cholangiocarcinoma, GBM, KIRC, acute myeloid leukemia (AML), LGG, and PAAD as compared to paired normal tissue (32). However, various malignancies had lower VISTA levels relative to paired normal tissues, including DLBCL and melanoma (32), which indicates that the impacts on VISTA expression can differ substantially in both magnitude and direction between malignancies. This heterogeneity in expression opens the possibility to consider VISTA expression as a potential biomarker for efficacy.

## Upregulation of VISTA expression after specific treatment modalities

VISTA expression has been measured on solid tumors after administration of various systemic therapies in an effort to identify potential resistance mechanisms. After neoadjuvant ipilimumab (anti-CTLA-4 monoclonal antibody [mAb]) plus androgen deprivation therapy, expression of VISTA and PD-L1 increased by 5-fold and 3-fold, respectively, on CD68+ macrophages in human localized prostate carcinomas (33). Immunofluorescence (IF) multiplex staining revealed that the majority of CD68+ macrophages either expressed PD-L1 (29.4%) or VISTA (26.5%), and only 2% expressed both (33). A murine KPC pancreatic cancer model showed after treatment with a DNA hypomethylating drug (decitabine), VISTA expression significantly increased from <5 cells/mm^2^ to > 15 cells/mm^2^ as assessed by IHC staining. Treatment with decitabine followed by anti-PD-1 or anti-VISTA therapy led to a further increase in CD8+ tumor-infiltrating lymphocytes (TILs) (34). When assessing for adaptive resistance in human melanomas, which initially responded then further progressed on either anti-CTLA-4 mAb + anti-PD-1 mAb (n=3) or anti-PD-1 mAb (n=13), the number of VISTA+ TILs increased in 67% of pretreatment–progression pairs (12 of 18). In contrast, an increased number of FoxP3+ regulatory T cells (Tregs) was seen in 56% (10 of 18) of progression biopsies with minimal increase in tumor cell PD-L1 expression. VISTA membranous expression was identified in immune cells in all biopsy specimens both within the tumor (intratumoral) and at the interface between the tumor and stroma/peri-tumoral region (35). While cytotoxic chemotherapies, including docetaxel, oxaliplatin, etoposide, 5-fluorouracil, gemcitabine, and cisplatin, have all been shown to upregulate PD-L1 on cancer cells and/or PD-1 on CD8+ TILs, it remains unknown whether VISTA expression is upregulated by cytotoxic chemotherapy (36).

## VISTA expression patterns in the tumor microenvironment

We conducted an extensive literature review of peer reviewed articles that assessed VISTA expression on specific cells and in specific compartments of malignant tumor tissues using IHC techniques. Many of these articles included expression of other checkpoint molecules (PD-1 and PD-L1) and CD8+ TILs, allowing several VISTA expression patterns to be defined.

### Myeloid/monocyte

VISTA is expressed on the myeloid/monocyte lineage in many tumors where VISTA expression is observed. In both non-small cell lung cancer (NSCLC) and pancreatic adenocarcinoma, VISTA is expressed on CD68+ tumor-associated macrophages (37, 38). VISTA expression was highest in CD68+ tumor-associated macrophages compared to other immune cell types in breast cancers but most prominent in the basal-like genotype (39).

For colorectal cancers, tumor-infiltrating macrophages (CD45+CD68+) expressed VISTA (40). In the peripheral blood of colorectal cancer, VISTA is mainly expressed by monocytic MDSCs (CD45+HLA-DR−CD14+) and monocytes (CD45+HLA-DR+CD14+). At the same time, intra-tumoral VISTA could be detected on almost all the tested subsets of myeloid cells. When comparing different subsets of myeloid cells in the blood and tumors, higher levels of VISTA expression were seen on all subsets in the tumors compared to peripheral blood, suggesting that TME contributes to VISTA expression, which in turn could promote tumor escape from antitumor immunity (40). It is known that increased VISTA expression can be driven by hypoxia, which may explain the heightened expression in the TME (41). After ipilimumab treatment for prostate cancer, there was a 5-fold increase in the expression of VISTA on CD68+ macrophages (33). In normal central nervous system (CNS) tissue, CNS myeloid cells (microglia and brain-border macrophages) express higher levels of VISTA than peripheral myeloid cells. VISTA expression is higher in microglia than in perivascular macrophages (20).

Additionally, VISTA is expressed on CD11b+ myeloid cells in the TME of melanoma, oral squamous cell carcinoma and in AML, where high expression of VISTA was detected on monocytes 46% (gated on CD45+CD11b+CD14hi/lo) and myeloid leukemia blasts 23% (gated by CD45int, CD45 vs. side scatter) and VISTA+ MDSCs 56% (42–44).

### T cells

VISTA expression can also be found on T cells in certain tumors (12, 38, 39, 45). In NSCLC, CD3+ infiltrating T cells are the most prominent immune cell expressing VISTA, with CD8+ cytotoxic cells expressing more VISTA than CD4+ T regulatory lymphocytes (38). In hepatocellular carcinoma (HCC), VISTA expression was significantly associated with CD8+ TILs (45). VISTA can be found on ~5% of CD4+ and CD8+ T cells within the TME of breast cancer (39). VISTA expression on circulating, non-tumoral T cells is also observed (46). VISTA expression level on tumor-infiltrating Tregs is higher than Tregs from peripheral lymph nodes, indicating that VISTA expressed on Tregs within the TME might play a role in suppressing tumor-specific immunity (47). In peripheral blood from patients who do not have malignancies, approximately 20% of CD4+ and 20% of CD8+ T cells show low-density VISTA staining (12). Minimal VISTA expression was observed on peripheral blood T cells (CD4+, CD8+ and Tregs) in AML patients (43).

### Malignant cells

VISTA can also be highly expressed on malignant cells themselves. Specific expression of VISTA on tumor cells measured by IHC regardless of tumor type ranges from 0 to 18% of tumor samples in most reports, with the highest percentage in mesothelioma (~90%), ovarian cancer (up to 28%), NSCLC (up to 20%), HCC (up to 16%) colon cancer (up to 15%), triple-negative breast cancer (11% to 18.5%) and gastric cancer (up to 9%) (31, 40, 45, 48–52).

### Mutual exclusivity

Expression of VISTA appears to be mutually exclusive between immune cells and tumor cells. In other words, tumors with VISTA on the tumor cell tend not to express VISTA on immune cells, and vice versa (45, 47, 53). Expression of VISTA on tumor cells was observed significantly more frequently among samples in which the immune cells were VISTA-negative (54). This may indicate that VISTA must only be expressed on a single cell population in the TME to have a local immune suppressive effect. An immune suppressive M2 macrophage gene signature was found to be correlated with *VISTA* mRNA expression in colorectal cancer (40).

## VISTA expression in the context of existing predictive markers

VISTA is expressed widely across the spectrum of malignancies and in diverse patterns with respect to other potential predictive markers (32).

### PD-L1

Tumors with PD-L1 expression are frequently (69–100%) also positive for VISTA expression (52, 54). This expression is predominantly on/in immune cells but is also present in nearly half of tumor cells. Others have shown a strong correlation between VISTA expression and PD-L1 expression (44). Nevertheless, significant numbers of tumors fall into four categories concerning VISTA and PD-L1 expression (PD-L1+/VISTA+, PD-L1+/VISTA–, PD-L1–/VISTA+ and PD-L1–/VISTA–) (48). Functionally PD-1 and VISTA appear to be non-redundant (55, 56). In fact, many PD-L1– tumors expressed VISTA; likewise, PD-L1+ tumors were negative for VISTA expression (51, 52, 57, 58).

Further understanding the relative expression of PD-L1 and VISTA and its implications for developing single or combination checkpoint blockade therapies will require more standardized methods for measuring and scoring VISTA expression through IHC. Currently, PD-L1 expression is determined by IHC, and specific scoring systems have been developed, considering the percentage of expression on tumor cells versus immune cells in the TME as a guide for anti-PD-1 and anti-PD-L1 therapies. A similar standardized system with defined scoring will also be needed to guide anti-VISTA therapies.

### TMB/MSI-high

TMB measures the number of mutations per [kB] using genomic sequencing. TMB with ≥10 mutations/megabase was recently found to be predictive of objective responses to pembrolizumab in various solid pediatric and adult tumors after prior therapy (59, 60) and was FDA-approved in June 2020 based on results from KEYNOTE-158 (NCT02628067) (61). Unfortunately, none of the papers reviewed assessed VISTA in the context of TMB. Microsatellite Instability (MSI) is a marker for defective DNA mismatch repair (dMMR), resulting in tumors accumulating thousands of mutations, most frequently in monomorphic microsatellites. A positive signal indicating MSI-High is determined when instability *via* PCR analysis is seen in two or more of the six mononucleotide repeat loci (NR-21, BAT-26, NR-27, BAT-25, NR-24, and MONO-27) (62). Positive finding with these markers is associated with improved outcome (63). Correlation between MSI+ colorectal cancer and VISTA has been observed by Zaravinos et al., with such tumors being highly sensitive to PD-1/PD-L1 blockade (64).

### CD8+

CD8+ T cells are critical mediators of antitumor immunity. The density of cytotoxic CD8+ and CD3+ T cells within the tumor and invasive margin of a tumor biopsy (called “ImmunoScore”) has emerged as a prognostic marker in patients with colon cancer (65). TIL density of >10% has independently been associated on multivariate analysis with better overall survival (hazard ratio [HR] = 0.48) and progression-free survival (HR = 0.40) in NSCLC treated with a PD-1 inhibitor (66). The presence of both CD8+ T cells and PD-L1 was predictive of response to durvalumab in advanced NSCLC compared with expression of either CD8+ T cells or PD-L1 expression alone (67).

There is some correlation between CD8+ TILs and VISTA expression, primarily in myeloid cells. Tumors with CD8+ ≥10% are twice as likely to be VISTA positive on immune cells and significantly correlate with a high density of CD8+ TILs (38, 45, 54). The expression of genes encoding VISTA was positively correlated with the expression of genes encoding CD8 (45, 52).

Significant subsets of tumors displayed a high number of CD8+ positivity and high expression of VISTA as assessed by IHC on tumor microarray analyses (44). In some cases VISTA expression was observed on lymphocytes (18, 27, 35, 40, 55, 68–71). VISTA expression has been shown to inhibit T-cell proliferation *in vitro* (27). Knockdown of VISTA or antibody inhibition of VISTA *in vitro* enhanced activation of CD8+ T cells. VISTA expression was associated with decreased numbers of CD8+ cells at the tumor site *in vivo* (27, 37, 41, 43). Alternatively, binding a VISTA-Fc fusion protein to surface Fc receptors has been shown to activate T cells (71).

### Regulatory T cells

Another effector T cell population, regulatory T cells (T regs), could be impacted by VISTA antagonism. Following a septic insult, investigators found that in wild-type mice, CD4+ T regs exhibit a significant upregulation of VISTA which correlates with higher T reg abundance in the spleen and small intestine. VISTA deficient mice have reduced T reg abundance in these compartments and a higher expression of Foxp3, CTLA4, and CD25 compared to wild-type mice. This suggests a protective T reg-mediated role for VISTA expression by which inflammation-induced tissue injury is suppressed and may lead to improved survival (72). In murine models, VISTA expression level on tumor-infiltrating Tregs is higher than Tregs from peripheral lymph nodes, indicating that VISTA expressed on Tregs within the TME might play a role in suppressing tumor-specific immunity. Additionally, VISTA blockade impaired the suppressive effect of Foxp3+/CD4+ regulatory T cells and the differentiation of tumor-specific peripheral Tregs (47).

### Neutrophil/MDSC

Neutrophil infiltrate dominates the immune cell composition in NSCLC and is inversely correlated with CD8+ and CD4+ T cells in the TME (73). Preclinical models in lung cancer demonstrated that high tumor-associated neutrophils correlated with resistance to PD-1 blockade, whereas neutrophil-depleting agents could reverse this phenomenon (74). Low frequencies of MDSC content in the TME appear to identify patients more likely to benefit from ipilimumab treatment (75).

Unlike PD-1 and CTLA-4, VISTA expression is prominent in myeloid cells, including neutrophils and MDSCs, which play a critical role in suppressing tumor-specific T-cell responses (8, 12); this suggests that VISTA plays a key role in tumor evasion from the immune system. Blocking VISTA has been shown to reduce MDSCs and neutrophils both *in vivo* and in patients (76). Thus, a potential predictive marker for VISTA efficacy may be VISTA-expressing neutrophils and/or MDSCs.

VISTA expression strongly correlates with the MDSC markers (CD11b and CD33) in HNSCC, AML, melanoma, pancreatic cancer, prostate cancer, esophageal cancer and colorectal cancer (33, 37, 41, 43, 44, 70, 77).

### IFN-γ signature

High IFN-γ mRNA expression was associated with improved median progression-free survival compared with low expression (5.1 vs. 2 months, HR = 6.66) (78). Researchers have shown that the patients with metastatic melanoma, HNSCC, and gastric cancer who responded to anti-PD-1 treatment had higher expression scores for IFN-γ-related genes when compared to non-responders. As a result, multiple IFN-γ signatures (e.g., IDO1, CXCL10, CXCL9, HLA-DRA, STAT1, and IFN–γ) have been proposed as predictive of the clinical response to immune CPIs (79). VISTA expression suppresses inflammatory cytokine secretion such as IFN-γ and TNF-α but also reduces the T-cell inflamed TME (28, 80).

This was observed in *in vitro* studies, where VISTA-Ig inhibited cytokine production, IL-2 primarily by CD4+ T cells and IFN-γ by CD8+ T cells (5). Additional *in vitro* studies showed that restimulation of PD-1 and VISTA double knockout T cells produced significantly higher concentrations of cytokines such as IFN-γ, TNF-α, and IL-17A compared to wild-type and single knockout PD-1 or VISTA T cells (56).

## Patterns of TME: VISTA positivity, PD-L1 positivity and CD8+ tumor-infiltrating lymphocytes

**Figure 3:**
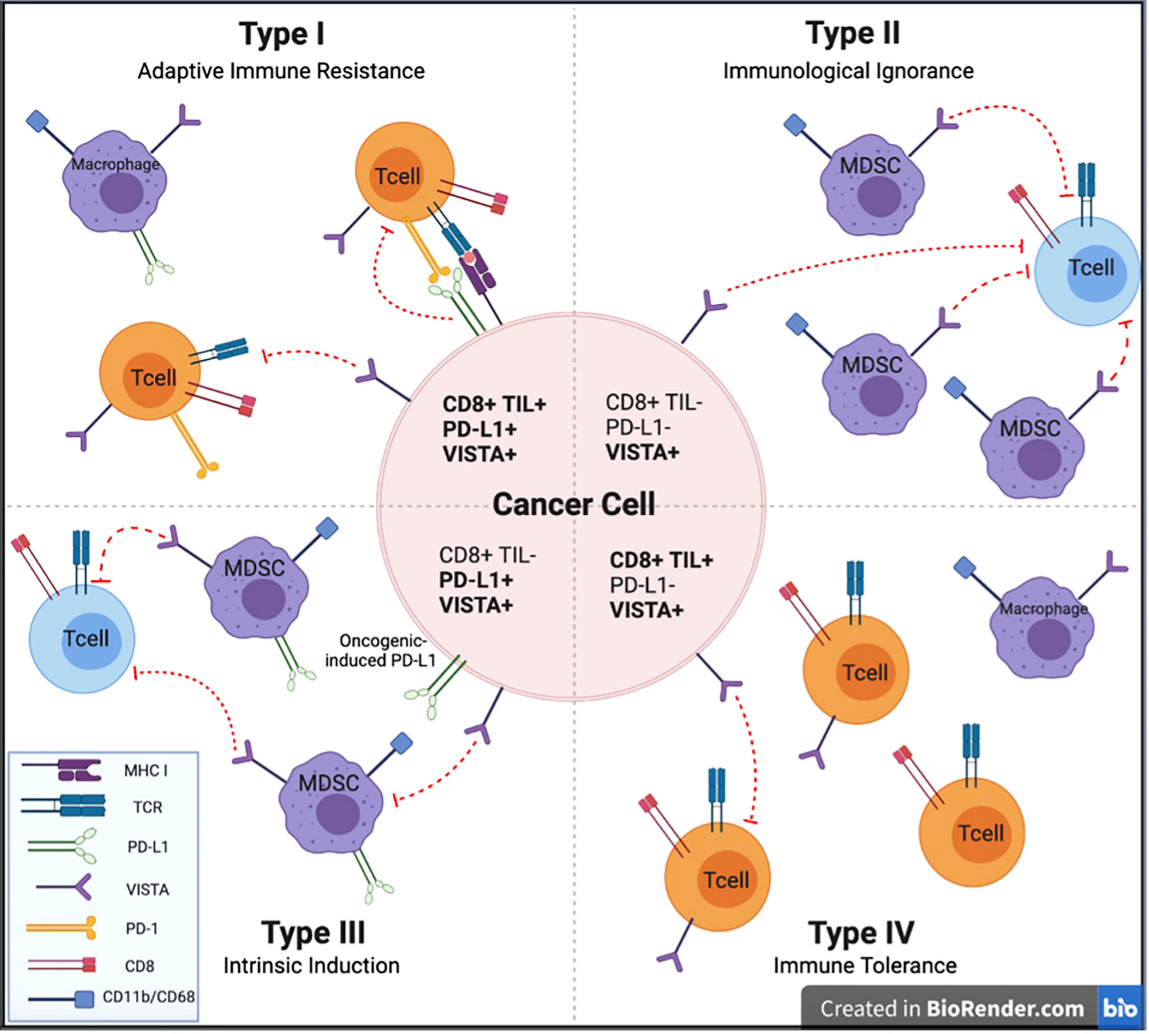
Types of tumor microenvironment targets for potential anti-cancer immunotherapies. Cancers are categorized into 4 different tumor microenvironments based on the presence or absence of CD8+ TILs and PD-L1 expression. We describe the potential impact of VISTA expression to each of these TME categories to facilitate investigation of anti-VISTA agents to improve upon immunotherapeutic strategies for each of these TME types. CD, Cluster of Differentiation; MDSC, myeloid derived suppressor cell; MHC I, major histocompatibility complex class I; PD-L1, programmed cell death ligand 1; PD-1, programmed cell death protein 1; TCR, T cell receptor; TIL, tumor-infiltrating lymphocyte; VISTA, V-domain Ig suppressor of T-cell activation.

Attempts to classify the immunogenicity of tumors have been based on immune cell-tumor cell interactions or lack thereof within the TME. Teng et al. described four distinct subgroups: Type I (PD-L1+ with TILs driving adaptive immune resistance), Type II (PD-L1– with no TILs indicating immune ignorance), Type III (PD-L1+ with no TIL indicating intrinsic induction), and Type IV (PD–L1– with TIL indicating the role of other suppressor(s) in promoting immune tolerance). Most melanomas present with a Type I or Type II TME at approximately 38% and 41%, respectively, with the resulting 20% having Type IV and 1% having Type III (81). VISTA is expressed in all of these different scenarios to different extents. **Figure 3** provides a schematic to illustrate the potential role of VISTA in each of these scenarios.

### Type I (TIL+; PD-L1+)-adaptive immune resistance

Adaptive immune resistance involves an inflamed TME with infiltrating lymphocytes that have become exhausted, resulting in PD-L1 expression. This pattern demonstrated the best response to anti-PD-1/PD-L1 therapy (82, 83). This pattern represents T-cell infiltration already in place, but their effectiveness is being suppressed by PD-1/PD-L1. This pattern has been described in coincidence with VISTA expression in the TME in HNSCC, pancreatic cancer, and NSCLC (37, 44, 53, 84). VISTA and PD-L1 are often mutually exclusive (52, 53). Separately, it has been documented that VISTA expression is strongly induced at the time of tumor progression following treatment with anti-PD-1 or anti-CTLA-4 therapy (33, 35). In this scenario, anti-VISTA therapy may contribute to anticancer activity in several ways, including lowering the threshold for activation of resting T cells and depleting immune suppressive MDSCs seen in poor-responding tumors to anti-PD-1/CTLA-4 therapy and activation of a cytotoxic NK-cell population (75). With VISTA maintaining a large percent of T cells in a quiescent state, anti-VISTA will bring these cells out of quiescence and lower their threshold for tumor antigen recognition, further enhancing T-cell effector function of antigen presentation on APCs promoted by anti-PD-1/PD-L1. MDSCs represent a powerful immune suppressive force driven by VISTA, and these can be depleted with anti-VISTA therapy. Finally, potent activation of the NK-cell population can be achieved by anti-VISTA blockade of VISTA signaling (76). Therefore, it is possible that anti-VISTA therapy will strongly augment anti-PD-1/PD-L1 therapy for Type I tumors when VISTA expression is present in the tumor. Anti-VISTA monotherapy may also be effective in this situation following progression on prior immunotherapy (47).

### Type 2 (TIL–; PD-L1–), immunological ignorance

A tumor lacking TILs and PD-L1 expression suggests a tumor that is not being recognized by the immune system. Patients with these tumors have traditionally had a poor prognosis and have largely not been responsive to single-agent immune checkpoint inhibition (30, 37, 69, 70). One treatment strategy of the combination of immune CPIs, such as anti-PD-1 and anti-CTLA-4, has been instituted to bring immune cells into the tumor compartment and prevent them from being suppressed on arrival. VISTA expression has been expressed on myeloid cells in the stromal compartment of these tumors. VISTA on MDSCs and other myeloid cells in the TME may contribute to this tumor phenotype by keeping T cells in a quiescent state, essentially raising the threshold for T-cell activation. Anti-VISTA therapy has increased infiltration of CD8+ and CD4+ T cells into the TME in a CT26 colon cancer mouse xenograft model, likely due to eliminating the inhibitory signals from VISTA on MDSCs (41). VISTA suppresses IFN response signaling, which also raises the T-cell threshold (85). Anti-VISTA therapy for VISTA-expressing tumors is expected to lower the threshold for T-cell activation by bringing these cells out of quiescence and enhancing antigen presentation on APCs. An example of tumors that frequently express an immunological ignorance phenotype is pancreatic ductal adenocarcinoma (PDAC) (37). Interestingly, VISTA is frequently expressed in these tumors, which may explain PDACs poor response to anti-PD-1, PD-L1, and CTLA-4 therapies (37, 69).

### Type 3 (TIL–; PD-L1+)-intrinsic induction

Intrinsic induction is a situation where PDL-1 is being expressed due to intrinsic tumor factors; however, no CD8+ TILs populate the tumor. Here PD-1-directed therapies would be less likely to be effective in the absence of T cells. VISTA expression in this setting has been described in multiple malignancies, including mesothelioma, gastric, HNSCC, renal cell and colorectal cancers, with expression most commonly observed in the TME on myeloid cells (20, 40, 48, 51, 57). VISTA+MDSCs may play a key role in preventing IFN signaling in the tumor. Additionally, VISTA expression on T cells outside of the TME may be critical for maintaining a T-cell-free TME by serving as the primary checkpoint holding circulating T cells in a quiescent state and unable to traffic into the tumor while also maintaining a high threshold for activation by APCs (7, 81).

### Type 4 (TIL+; PD-L1–)-tolerance/other suppressors

Immune tolerance is a situation where T cells are present, but some other mechanism suppresses their antitumor effectiveness. VISTA inhibitory function, possibly in combination with other suppressive pathways, has been attributed to this immunosuppressive state (81). MDSCs, which are regulated by VISTA, represent a strong immune suppressive signal that directly impairs the functioning of effector T cells. Tumors with cells with this tolerance phenotype with strong VISTA expression have been described in various malignancies, including ovarian cancer and HCC (45, 86). They may significantly benefit from targeting VISTA (52).

## Summary of mechanisms of VISTA function in the setting of cancer

**Figure 4:**
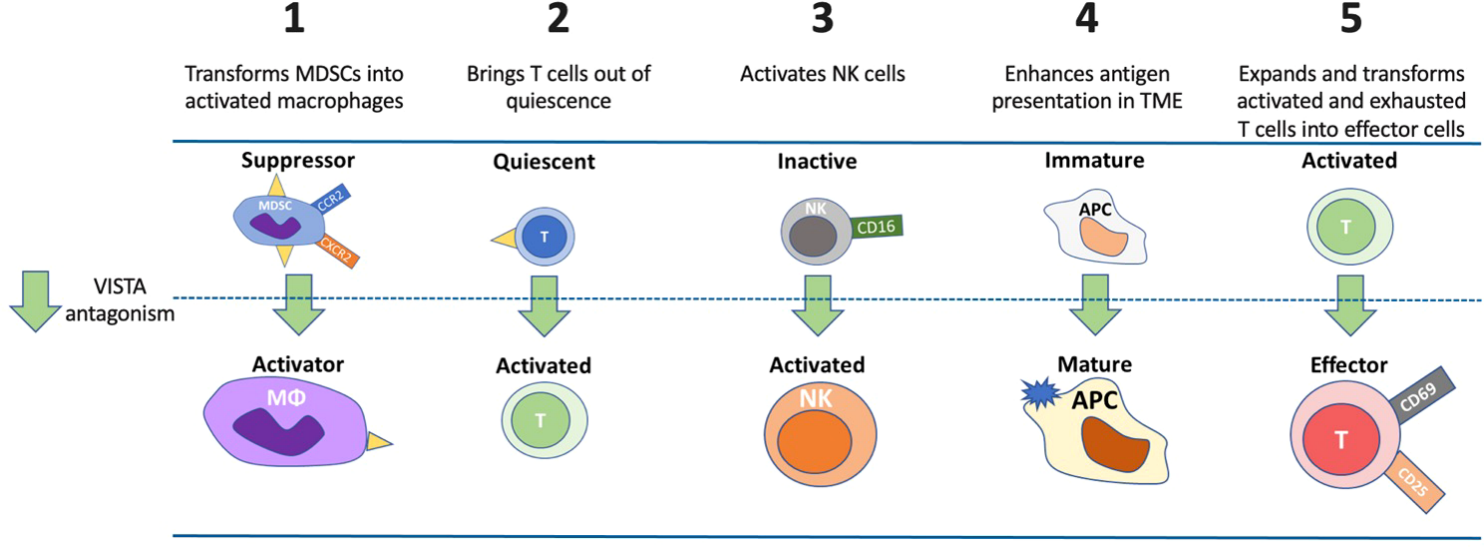
Potential mechanisms and impacts of VISTA antagonism. VISTA plays multiple roles in controlling immune responses. VISTA antagonism 1) induces a shift in MDSC function, reduces MDSC suppressive activity and enhances the potential for improved antitumor effect through macrophage activation, 2) lowers the threshold of activation for quiescent T cells and allows a greater percentage of T cells to respond to neo-antigens, 3) activates NK cells that may result in independent anticancer effects *via* antibody-directed cellular cytotoxicity, 4) enhances APC maturation to effectively present antigens to induce a T-cell response, and 5) expands and transforms activated and exhausted T cells into effector cells. APC, antigen presenting cell; CCR2, C-C motif chemokine receptor; CD, cluster of differentiation; CXCR2, CXC motif chemokine receptor 2; MDSC, myeloid-derived suppressor cell; MΦ, macrophage; NK, natural killer cell.

VISTA plays multiple key roles in controlling immune responses, several of which are unique relative to other immune checkpoint molecules. **Figure 4** illustrates these mechanisms and the effect of VISTA antagonism. First, VISTA is a dominant checkpoint on MDSCs (41), and these cells exert a powerful suppressive influence on effector T cells (87). VISTA antagonism induces a shift in MDSC function, reducing their immune suppressive activity and enhancing the potential for improved antitumor effects (47).

A second cancer-related mechanism of VISTA is maintaining T cells in a quiescent state, where VISTA is the dominant immune checkpoint molecule expressed in these cells (7). In this state, the threshold for activation of T cells by antigen is extremely high. VISTA antagonism of these quiescent T cells significantly lowers this threshold, allowing a greater percentage of T cells to respond to self- and cancer-derived neo-antigens (7).

A third cancer-related mechanism linked to anti-VISTA therapy is the FcR-dependent activation of NK cells. Activation of NK cells is observed upon VISTA inhibition and may result in independent anticancer effects *via* antibody-directed cellular cytotoxicity. Similarly to keeping T cells in a quiescent state, VISTA could also contribute to maintaining NK cells in an inactive state.

A fourth anticancer mechanism of anti-VISTA is maintaining APCs in an immature, non-inflammatory state. In this state, APCs are impaired in their ability to present antigens effectively. Upon VISTA inhibition, APCs undergo maturation and can more effectively present antigens to induce a T-cell response (5). This is seen in the setting of renal cell carcinoma, where VISTA is mainly upregulated in APCs (CD14+HLA-DR+ macrophages), and VISTA inhibition was associated with decreased tumor growth (57).

A fifth anticancer mechanism of anti-VISTA involves the transition of activated T cells into effector T cells. A variety of cytokines are required for this transition, and these are negatively regulated by VISTA. This phenomenon has been demonstrated in graft versus host disease mouse models as VISTA signaling through a VISTA-agonist-antibody has been shown to result in direct suppression of effector T cells rather than direct effects on Treg cells with concomitant reduction in IFN-γ and TNF-α levels in CD4+ and CD8+ T cells (88). Positively triggering VISTA signaling using anti-VISTA agonistic antibodies or other VISTA ligands may have implications for treating autoimmune disease.

Finally, while VISTA is prominently expressed in resting T cells, it is also expressed in activated and memory T cells (89). The antagonism of VISTA on memory T cells will likely enhance antitumor immunity, although this needs to be confirmed through further experimentation. In summary, VISTA plays multiple roles that directly affect immune function in the setting of cancer and VISTA may play a dominant role in several of these situations relative to other immune checkpoint regulators.

## Safety implications of targeting VISTA

The potential benefits of anti-tumor efficacy using anti-VISTA therapy (**Figure 4**) through the multiple anti-cancer mechanisms described above must be evaluated in the context of the risks of immune system stimulation, which could conceivably be amplified when combined with other checkpoint inhibitors (anti-CTLA-4 and anti-PD-1/PD-L1) already known to induce the immune system. However, unlike the lethal phenotype seen in CTLA-4 knockout mice, mice that lack VISTA do not develop severe systemic autoimmune pathology, suggesting that other immune-regulatory pathways mitigated the development of inflammation and autoimmunity induced by VISTA deficiency (85). VISTA is important for maintaining peripheral tolerance through T-cell quiescence, so blocking VISTA might decrease peripheral tolerance and promote development of auto-inflammatory conditions. In particular, the broad expression of VISTA on myeloid cells, including MDSCs, may lead to systemic consequences for inhibiting VISTA that extend beyond the TME. Cytokine release is another concern which has been demonstrated in a mixed leukocyte reaction (MLR) of human peripheral blood mononuclear cells (PBMCs) with multiple anti-VISTA monoclonal antibodies which is dependent on them having functional Fc domains (90). Cytokine release with peak levels of cytokines between 2-6 hrs has been seen with Fc-active-anti-VISTA blockade in a human early phase trial which investigators will need to mitigate through dosing adjustments and additional anti-inflammatory medications (76).

## Conclusions and future prospects

VISTA is a novel immune checkpoint regulator molecule with unique expression patterns within the TME relative to other key immune checkpoint molecules. VISTA is emerging to play key roles in various immunoregulatory mechanisms in patients with cancer, including adaptive immune resistance, immunological ignorance, intrinsic induction, and tolerance. VISTA is found in multiple cell types, including myeloid cells, lymphoid cells, and in tumor cells themselves. Certain patterns are starting to emerge with differential VISTA expression on different cell types within the TME and the different conditions under which VISTA expression is induced. The interplay between VISTA and the components of the TME may be central in predicting the efficacy of anti-VISTA agents currently under development, as well as in the optimal use of these antagonists in combination with other immune-modulating therapies.

VISTA is emerging along with other markers, such as PD-1, PD-L1 and CTLA-4, together with other factors (e.g., TMB and microsatellite instability) to define several specific immunological states in the TME. Four distinct patterns implicating VISTA along with these other markers can be defined where VISTA can contribute to immunoregulation; this may inform when and where to deploy therapies impinging on VISTA function alone or in combination with other immunotherapies. An individualized approach to selecting therapies for solid tumors based on these TME sub-types (shown in **Figure 2**) and other similar frameworks could significantly impact clinical treatment and improve patient-related outcomes. Greater understanding of VISTA’s role in each scenario can form the basis for selecting or enriching patients for specific therapies and therapeutic combinations where complementing PD-1/PD-L1 or CTLA-4 interactions by targeting VISTA could hold great promise to improve clinical responses. For example, there is evidence that anti-CTLA-4 and anti-PD-1 therapy results in the upregulation of VISTA in patients with localized prostate adenocarcinoma and metastatic melanoma during treatment forming a basis to test such combination therapies (33, 35).

VISTA is a compelling treatment target, and clinical trials have been initiated to evaluate 3 anti-VISTA antibodies and one oral antagonist (**Table 1**); all three antibodies are currently being assessed in phase 1 active trials listed. To date, agents targeting VISTA have been administered as monotherapies, but several trials aim to assess outcomes together with anti-PD-1 antibodies. It will be important that these and future human trials of anti-VISTA monoclonal antibodies and small molecule VISTA inhibitors include correlative studies on which different TME landscapes are most responsive to therapy; this will require biomarker-focused clinical trials with tumor biopsies before and after treatment.

**Table 1:** Clinical studies of agents targeting the VISTA checkpoint.

Intervention	MOA	Condition(s)	Study description	Enrolled (N)	Identifier	Sponsor	Location	Status
JNJ-61610588 (CI-8893)	Fully human IgG1ĸ Anti-VISTA mAb	Advanced cancers	An open-label, first-in-human Phase 1 study to evaluate the safety and tolerability of JNJ-61610588 in adults	12	NCT02671955	Janssen Research & Development, LLC	USA	Terminated
CI-8993	Fully human IgG1ĸ Anti-VISTA mAb	Advanced solid tumors (non-lymphoma)	A multi-center, open-label, Phase 1 dose-escalation study to determine the maximum tolerated dose of CI-8993 in adult patients	16	NCT04475523	Curis, Inc.	USA	Recruiting
CA-170	VISTA/PD-L1/ PD-L2 antagonist	Advanced solid tumors or lymphoma	A multi-center, open-label, Phase 1 study of orally administered CA-170 in adult patients	71	NCT02812875	Curis, Inc.	USA	Completed
HMBD-002	Fc-independent IgG4 Anti-VISTA mAb	Advanced solid tumors	A multi-center, open-label, first-in-human, Phase 1/2 study evaluating multiple doses of HMBD-002, with or without pembrolizumab, in adult patients with solid tumors	Unknown	NCT05082610	Hummingbird Bio, Inc.	USA	Recruiting
W0180	Anti-VISTA mAb	Locally advanced or metastatic solid tumors	A multi-center, open-label, first-in-human, Phase 1 dose escalation and dose expansion study evaluating W0180 as a monotherapy and in combination with pembrolizumab in adults with locally advanced or metastatic solid tumors	Unknown	NCT04564417	Pierre-Fabre	France/Spain	Active, not recruiting
KVA12123 (KVA12.1)	Fully human IgG1 Anti-VISTA mAb	Advanced solid tumors	Kineta is planning to conduct a Phase 1/2 study evaluating KVA12123 as a monotherapy and in combination with pembrolizumab in patients with advanced solid tumors	NA	NA	Kineta, Inc.	USA	Planned

IgG, immunoglobulin; mAb, monoclonal antibody; NA, not applicable; PD-L, programmed death-ligand; VISTA, V-domain immunoglobulin suppressor of T cell activation.

Further research will be needed to tease out the role of VISTA on NK cells and other innate lymphoid cells and how VISTA affects dendritic cells in both the TME and tumor-draining lymph nodes. Similar to PD-1/PD-L1 blockade, which has been shown to activate tumor-specific T cells in tumor-draining lymph nodes, the expression and role of VISTA in secondary lymphoid organs needs to be better defined. In addition, there will be a need to develop standardized reagents to measure and score VISTA expression in tumors together with other immune checkpoints, such as PD-L1. Patient selection for anti-VISTA therapies will eventually require standardized tests that score the extent and location of VISTA expression.

## Author contributions

AM and RM were responsible for manuscript conception and design; data collection, analysis, and interpretation; and drafting and critical revision of the manuscript. MM, AU, RV, RN, LL, MJ, and LR provided critical revision of the manuscript. All authors contributed to the article and approved the submitted version.
